# Tumor necrosis factor- α, adiponectin and their ratio in gestational diabetes mellitus

**DOI:** 10.22088/cjim.9.1.71

**Published:** 2018

**Authors:** Ali Khosrowbeygi, Mohammad Reza Rezvanfar, Hassan Ahmadvand

**Affiliations:** 1 Department of Biochemistry and Genetics, School of Medicine, Arak University of Medical Sciences, Arak, Iran.; 2 Department of Internal Medicine, Arak University of Medical Sciences, Arak, Iran.; 3 Department of Biochemistry, School of Medicine, Lorestan University of Medical Sciences, Khorramabad, Iran.

**Keywords:** Gestational diabetes mellitus, Adiponectin, Tumor necrosis factor- α, Insulin resistance

## Abstract

**Background:**

It has been suggested that inflammation might be implicated in the gestational diabetes mellitus (GDM) complications, including insulin resistance. The aims of the current study were to explore maternal circulating values of TNF-α, adiponectin and the adiponectin/TNF-α ratio in women with GDM compared with normal pregnancy and their relationships with metabolic syndrome biomarkers.

**Methods:**

Forty women with GDM and 40 normal pregnant women were included in the study. Commercially available enzyme-linked immunosorbent assay methods were used to measure serum levels of TNF-α and total adiponectin.

**Results:**

Women with GDM had higher values of TNF-α (225.08±27.35 vs 115.68±12.64 pg/ml, p<0.001) and lower values of adiponectin (4.50±0.38 vs 6.37±0.59 µg/ml, p=0.003) and the adiponectin/TNF-α ratio (4.31±0.05 vs 4.80±0.07, P<0.001) than normal pregnant women. The adiponectin/TNF-α ratio showed negative correlations with insulin resistance (r=-0.68, p<0.001) and triglyceride (r=-0.39, p=0.014) and a positive correlation with insulin sensitivity (r=0.69, p<0.001). Multiple linear regression analysis showed that values of the adiponectin /TNF-α ratio were independently associated with insulin resistance. Binary logistic regression analysis showed that GDM was negatively associated with adiponectin /TNF-α ratio.

**Conclusions:**

In summary, the adiponectin/TNF-α ratio decreased significantly in GDM compared with normal pregnancy. The ratio might be an informative biomarker for assessment of pregnant women at high risk of insulin resistance and dyslipidemia and for diagnosis and therapeutic monitoring aims in GDM.

Gestational diabetes mellitus (GDM), which is defined as glucose intolerance with onset or first diagnosis during pregnancy, is one of the most important pregnancy complications (1-4). It affects approximately 1 to 20% of all pregnancies worldwide and its prevalence in Iranian women is about 4 to 9% of all pregnancies (5). It has been suggested that inflammation might be implicated in the GDM pathogenesis (6). In recent years, the role of pro-inflammatory cytokines such as tumor necrosis factor- α (TNF-α), and anti-inflammatory cytokine such as adiponectin has been increasingly studied in GDM (4). TNF-α is one of proposed molecules that can cause insulin resistance during pregnancy. On the other hand, adiponectin has been suggested as the strongest anti-inflammatory cytokine that promotes insulin sensitization by stimulating AMP-activated protein kinase that leads to increase glucose uptake in skeletal muscle (7, 8). There are some conflicting findings of maternal circulating levels of the cytokines in women with GDM compared with normal pregnancy (5, 7). 

Therefore, the aims of the current study were to explore maternal circulating values of TNF-α, adiponectin and the adiponectin/TNF-α ratio in Iranian women with GDM compared with normal pregnancy and their relationships with metabolic syndrome biomarkers. 

## Methods

This case-control study was conducted at the Obstetrics and Gynecology Hospital of Lorestan University of Medical Sciences (July, 2014- March, 2015) after being approved by the Institutional Ethics Review Board. Informed consent was obtained from each pregnant woman enrolled in this study. The study population consisted of 40 nulliparous women with newly diagnosed GDM before any drug treatment, and 40 normal nulliparous pregnant women at 24-28 weeks of gestation. All subjects were Iranian with Lor ethnicity. The diagnosis of GDM was made according to the 75-g oral glucose tolerance test (OGTT) (9). Exclusion criteria were smoking, multiparity, diabetes mellitus, chronic hypertension, preeclampsia and the patients who were under treatment with metformin or insulin (10).

Commercially available photometric methods were used to measure fasting serum levels of glucose (FBG), total cholesterol (TC), triglyceride (TG) (ZiestChem Diagnostic, Tehran, Iran) and high-density lipoprotein cholesterol (HDL-C) (Parsazmun, Karaj, Iran) using a chemistry analyzer (Hitachi, Germany). The intra-assay and inter-assay coefficients of variation were <10% according to the manufacturers. Low-density lipoprotein cholesterol (LDL-C) was estimated by Friedewald’s equation (11). The lipid ratios including the TC/HDL-C ratio, the LDL-C/HDL-C ratio and the TG/TC ratio were calculated (12). The atherogenic index of plasma (AIP) was calculated using log (TG/HDL-C) formula (13). 

Commercially available enzyme-linked immunosorbent assay (ELISA) methods were used to measure serum levels of TNF-α (Ani Biotech Oy, Orgenium Laboratories Business Unit, Finland), total adiponectin (BioVendor Laboratory Medicine, Inc. Czech Republic) and insulin (Monobind Inc., USA) using an ELISA reader (STAT FAX 3200, USA). The intra-assay coefficient of variation of all the ELISA assays was <10% according to the manufacture. The sensitivities of the assays were 15 pg/ml, 26 ng/ml and 0.75µIU/ml, respectively, according to the manufacture. The adiponectin/TNF-α ratio was calculated and log-transformed. Insulin resistance was calculated using the homeostasis model assessment of insulin resistance (HOMA-IR) index formula (14). The quantitative insulin sensitivity check index (QUICKI) was used to estimate insulin sensitivity (15).

The Kolmogorov–Smirnov test was used to explore the normality of distribution of the variables. The Student t-test and Pearson’s correlation analysis were used to do statistical calculations for quantitative variables with normal distributions. Skewed parameters were analyzed by the Mann–Whitney U-test and Spearman’s correlation. Multiple linear regression analysis was also performed to determine the independent predictors of FBG, the HOMA-IR index and the QUICKI as the dependent variables. Non-normally distributed parameters were log-transformed for the multiple linear regression analysis. To assess the risk markers for the diagnosis of GDM, a multiple logistic regression analysis was performed. A *p*-value less than 0.05 was considered statistically significant. Data are presented as mean±SEM. Statistical computations were done using SPSS Version 19 software.

## Results

The demographic and biochemical characteristics of women with GDM and normal pregnant women are presented in tables 1 and 2. There were no significant differences in gestational age (26.25±0.24 vs 26.13±0.22 weeks, p=0.672) and BMI (27.25±0.18 vs 27.66±0.17 kg/m^2^, p=0.107) between normal pregnant women and patients. Though, women with GDM were older than control group (31.67±0.54 vs 29.15±0.58, p=0.002). 

Women with GDM had higher values of FBG (100.83±1.54 vs 78.45±1.29 mg/dl, p<0.001), the LDL-C/HDL-C ratio (5.17±0.58 vs 3.23±0.36, p=0.014), the TC/HDL-C ratio (7.88±0.65 vs 5.32±0.54, p<0.001), the TG/TC ratio (1.17±0.10 vs 0.94±0.06, P=0.046), AIP (0.87±0.04 vs 0.63±0.04, p<0.001), insullin (12.56±0.90 vs 9.54±0.55 µIU/ml, P=0.006), the HOMA-IR index (3.11±0.22 vs 1.85±0.11, p<0.001) and TNF-α (225.08±27.35 vs 115.68±12.64 pg/ml, p<0.001) than normal pregnant women. Although, values of QUICKI (0.33±0.01 vs 0.36±0.01, p<0.001), adiponectin (4.50±0.38 vs 6.37±0.59 µg/ml, P=0.003) and the adiponectin/TNF-α ratio (4.31±0.05 vs 4.80±0.07, p<0.001) were significantly lower in GDM than in normal pregnancy. Comparisons of the values of TNF-α and adiponectin between women with GDM and normal pregnant women remained statistically significant after adjusting for maternal age.

**Table 1:** Demographic characteristics of women with gestational diabetes mellitus (GDM) compared with normal pregnant women

**Parameter**	**Normal pregnant** ** (n=51)**	**GDM** **(n=49)**	**Pvalue**
Age (years)	29.15±0.58	31.67±0.54	0.002
Gestational age(weeks)	26.25±0.24	26.13±0.22	0.672
BMI (kg/m^2^)	27.25±0.18	27.66±0.17	0.107

Data are presented as mean ± SEM. BMI = body mass index.

**Table 2:** Biochemichal characteristics of gestational diabetes mellitus (GDM) and normal pregnant women

**Parameter**	**Normal pregnant** **(n=40)**	**GDM** **(n=40)**	**Pvalue**
FBG (mg/dl)	78.45±1.29	100.83±1.54	<0.001
TG (mg/dl)	221.83±14.00	256.12±19.12	0.236
TC (mg/dl)	242.67±10.18	225.84±9.88	0.239
HDL-C (mg/dl)	52.21±2.95	34.70±2.71	<0.001
LDL-C (mg/dl)	146.10±7.84	139.92±10.11	0.341
TG/TC ratio	0.94±0.06	1.17±0.10	0.046
TC/HDL-C ratio	5.32±0.54	7.88±0.65	<0.001
LDL-C/HDL-C ratio	3.23±0.36	5.17±0.58	0.014
AIP	0.63±0.04	0.87±0.04	<0.001
Insullin (µIU/ml)	9.54±0.55	12.56±0.90	0.006
HOMA-IR	1.85±0.11	3.11±0.22	<0.001
QUICKI	0.36±0.01	0.33±0.01	<0.001
Adiponectin (µg/ml)	6.37±0.59	4.50±0.38	0.003
TNF-α (pg/ml)	115.68±12.64	225.08±27.35	<0.001
Adiponectin /TNF-α ratio	4.80±0.07	4.31±0.05	<0.001

Data are presented as mean±SEM.

FBG= fasting blood glucose, TG= triglyceride, TC= total cholesterol, HDL-C= high-density lipoprotein cholesterol, LDL-C= low-density lipoprotein cholesterol, AIP= atherogenic index of plasma, HOMA-IR= homeostasis model assessment of insulin resistance, QUICKI= quantitative insulin sensitivity check index, TNF-α= tumor necrosis factor- α

**Figure 1:**
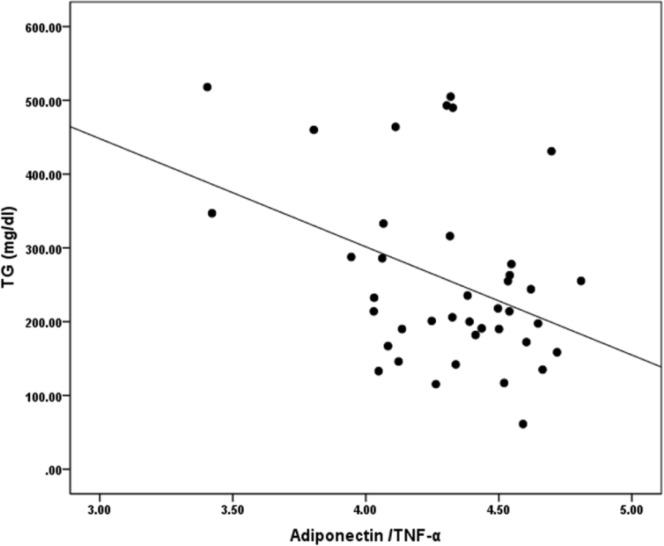
Correlations between values of TG and the adiponectin /TNF-α ratio in women with gestational diabetes mellitus (r=-0.39, p=0.014

The HOMA-IR index values correlated negatively with values of adiponectin (r=-0.45, p=0.004) and the adiponectin/TNF-α ratio (r=-0.68, p<0.001) and positively with the levels of TNF-α (r=0.60, p<0.001) in normal pregnant women (data not shown). The QUICKI showed positive correlations with values of adiponectin (r=0.45, p=0.004) and the adiponectin/TNF-α ratio (r=0.69, p<0.001) and negative correlations with values of TNF-α (r=-0.59, p<0.001), the LDL-C/HDL-C ratio (r=-0.31, p=0.048) and the TG/TC ratio (r=-0.33, p=0.036) in women with normal pregnancy (data not shown). In GDM, values of FBG correlated negatively with adiponectin (r=-0.34, p=0.033) (data not shown). Values of TG correlated positively with the HOMA-IR index (r=0.35, p=0.027) and negatively with the QUICKI (r=-0.34, p=0.034), adiponectin (r= -0.42, P=0.007) (data not shown) and the adiponectin/TNF-α ratio (r=-0.39, p=0.014) (fig. 1) in women with GDM. The TG/TC ratio values correlated negatively with adiponectin (r=-0.43, p=0.006) and the adiponectin/TNF-α ratio (r=-0.48, p=0.002) in GDM (data not shown). 

The correlations were also examined in total subjects of entire GDM and normal pregnant women (n=80). Levels of adiponectin correlated negatively with values of TNF-α (r=-0.24, p=0.030), TG (r=-0.35, p=0.002), and the TG/TC ratio (r=-0.37, P=0.001) in the pooled sample (data not shown). Adiponectin showed slightly negative correlation with AIP (r=-0.21, p=0.065). Values of the adiponectin/TNF-α ratio showed negative correlations with values of TG (r=-0.24, p=0.030) and the TG/TC ratio (r=-0.29, p=0.011) in the pooled sample (data not shown). In the total sample, serum levels of FBG correlated positively with values of TNF-α (r=0.51, p<0.001), TG (r=0.22, p=0.048), the TG/TC ratio (r=0.26, p=0.018), the TC/HDL-C ratio (r=0.32, p=0.003) and AIP (r=0.44, p<0.001) and negatively with values of HDL-C (r=-0.40, p<0.001), adiponectin (r=-0.39, p<0.001) and the adiponectin/TNF-α ratio (r= -0.54, p<0.001) (data not shown).

 The HOMA-IR index showed positive correlations with values of TNF-α (r=0.38, p=0.000), TG (r=0.30, P=0.006), the TG/TC ratio (r=0.35, p=0.001) and AIP (r=0.36, p=0.001) and negative correlations with values of adiponectin (r=-0.38, p<0.001) and the adiponectin/TNF-α ratio (r=-0.45, p<0.001) in the pooled sample (data not shown). Values of QUICKI correlated negatively with values of TNF-α (r=-0.38, p=0.001), TG (r=-0.31, P=0.006), the TG/TC ratio (r= -0.35, p=0.001) and AIP (r=-0.36, p=0.006) and positively with values of adiponectin (r=0.38, p<0.001) and the adiponectin/TNF-α ratio (r=0.45, p<0.001) in the pooled sample (data not shown). 

Multiple linear regression analysis with FBG, the HOMA-IR index and the QUICKI as the dependent variables and all the variables that showed statistically significant correlations with them in the univariate analysis were also performed. In this model, values of the adiponectin /TNF-α ratio were independently associated with FBG (β=- 0.46, p<0.001), the HOMA-IR index (β=- 1.54, p=0.003) and the QUICKI (β=0.37, p=0.001).

 The risk markers for the diagnosis of GDM were assessed using binary logistic regression analysis. Univariate binary logistic regression analysis for lipid profile, the lipid ratios, adiponectin, TNF-α and the adiponectin/TNF-α ratio showed that the LDL-C/HDL-C ratio, the TC/HDL-C ratio, AIP and TNF-α were positive risk markers for GDM development while HDL-C, adiponectin and the adiponectin/TNF-α ratio might have protective effects. A multiple binary logistic regression analysis with the lipid ratios, AIP and HDL-C showed that AIP was indepently a risk marker for GDM development.

 A multiple binary logistic regression analysis for adiponectin, TNF-α and the adiponectin/TNF-α ratio showed that the ratio was indepently a negative risk marker for GDM development. A multiple binary logistic regression analysis for AIP and the adiponectin/TNF-α ratio showed that AIP was indepently a risk marker for GDM development while the adiponectin/TNF-α ratio had protective effects (table 3). 

**Table 3:** Multiple binary logistic regression analysis of the effects of AIP and Log (adiponectin /TNF-α) on gestational diabetes mellitus.

**Variables**	**B**	**OR**	**95% CI for OR**	**pvalue**
AIP	3.67	39.18	3.963–387.363	0.002
Adiponectin /TNF-α ratio	- 4.72	0.009	0.001–0.122	<0.001

CI confidence interval, OR, odds ratio, Other abbreviations are given in Table 1

## Discussion

The main findings of the present study were a significantly lower level of the adiponectin/TNF-α ratio in GDM compared with normal pregnancy and significant associations of the ratio with metabolic syndrome biomarkers. There are some conflicting findings of maternal circulating levels of the cytokines adiponectin and TNF-α in women with GDM compared with normal pregnant women and their associations with metabolic syndrome biomarkers. López-Tinoco et al. (16) and Liu et al.’s (17) studies showed that levels of adiponectin decreased and levels of TNF-α increased significantly in GDM compared with normal pregnancy. In Liu et al.’s study, the HOMA-IR index values have been negatively associated with adiponectin and positively with TNF-α. (17). These findings were confirmed by the current study. On the other hand, Kim et al. showed that the maternal serum levels of total adiponectin in GDM are significantly lower than normal pregnancy, while the levels of TNF-α do not change significantly between two groups (18). 

In another study in Tunisia, women with GDM have higher values the HOMA-IR index and lower levels of adiponectin than normal pregnant women, but levels of TNF-α do not differ significantly between two groups (19). A study in Iranian pregnant women showed that values of adiponetin, the TG/HDL-C ratio and QUICKI do not change significantly between GDM and normal pregnancy while HOMA-IR index values increased in GDM (5). A systematic review study by Gomes et al. reported that women with GDM in the 3rd trimester of pregnancy have slightly higher values of TNF-α than normal pregnancy. They proposed that several factors, including gestational age, ethnicity, smoking and BMI could influence cytokine production during pregnancy. Therefore, patients and normal control women should be matched for BMI and gestational age and their smoking habits and ethnicity should be clarified in the studies (6). It has been proposed that adiponectin has anti-inflammatory properties by suppressing TNF-α gene transcription (20). The indirect correlation observed between these cytokines in the current study might be addressed by this proposed mechanism. It has also been proposed that TNF-α increasing in GDM can suppress transcription of adiponectin that results in insulin resistance and hyperinsulinemia (21). Therefore, significantly decreased adiponectin/TNF-α ratio could lead to some important metabolic complications that were shown in the current study. 

The ratio showed negative correlations with insulin resistance, TG and the TG/TC ratio and positive correlation with insulin sensitivity in the current study. Although many important physiological functions have been addressed to adiponectin, it is not vital to life under normal conditions. Desoite that the role of the cytokine will be essential under hyperglycemic and dyslipidemic situations. On the other hand, certain diabetes drugs that decrease insulin resistance only work in the presence of the cytokine. Systemic highly lipotoxic environment due to severe hyperlipidemia –the common process in types 1 and 2 diabetes –is toxic for beta cells. Adiponectin stimulate lipid storage in adipocytes which may contribute, at least in part, to the reduction of beta cells lipotoxicity (22). 

Study of Li et al. showed that adipose tissue partly regulates hepatic cholesterol metabolism via adiponectin. They developed a co-culture system of adipocytes and hepatocytes to study the interaction between these two cell types. They observed that adiponectin can decrease activity of 3-hydroxy-3-methyl-glutaryl-CoA reductase ( HMGR), a key enzyme in cholesterol biosynthesis pathway, and increase expression of ATP-binding cassette transporter A1 (ABCA1) which plays a crucial role in the efflux of cellular cholesterol to HDL (23). 

An intervention study conducted by Aye et al. in a mouse model of obesity in pregnancy shows that using synthetic adiponectin receptor agonist may be a helpful intervention treatment to increase functional adiponectin on maternal and placental tissues to reduce the metabolic and hormonal complications due to maternal obesity and GDM (24). It has been shown that, in contrast to adiponectin, TNF-α stimulates amino acid transport in primary human trophoblasts through system A transporter (25). Thus, normalization of adiponectin can attenuate TNF-α activity. 

According to the available data, it could be concluded that adiponectin might be the most important cytokine in the regulation of inflammatory and metabolic syndrome status in GDM. An indirect correlation observed between adiponectin and TNF-α in the current study could be addressed that hypoadiponectinemia in GDM results in increased levels of TNF-α (21). Adiponectin infusion in pregnant mice results in a significant decrease in maternal values of leptin and insulin (24). Hence, hypoadiponectinemia leads to hyperleptinemia and hyperinsulinemia that could be addressed as increased insulin resistance. Consequently, the negative correlations of adiponectin and the adiponectin/TNF-α ratio with FBG and TG that observed in the current study could be addressed as the protective effects of adiponectin against metabolic syndrome complications in women with GDM. Adiponectin decreases placental system A activity that leads to decreasing amino acid transportation to fetus. As a consequenc, adiponectin attenuates the effects of TNF-α and interlukin-10 on fetus (25). On the contrary the adiponectin/TNF-α ratio decreased in GDM and showed a negative correlation with insulin resistance. 

The major limitations of the current study were: (i) small sample size; (ii) the groups did not match for age; and (iii) the study designed as a case-control should be designed as a cohort study for exploring the adiponectin/TNF-α ratio importance as a possible marker in GDM.

In conclusion, the adiponectin/TNF-α ratio might be more informative than diponectin and TNF-α alone for predicting women with high risk of insulin resistance and metabolic syndrome in pregnancy. The adiponectin /TNF-α ratio could be introduced as a possible negative risk factor of GDM that might be useful for diagnosis and/or therapeutic monitoring aims. Nonetheless, further cohort studies with higher sample size might be required for exploring the ratio importance in GDM. 

## References

[1] Yeral MI, Ozgu-Erdinc AS, Uygur D (2014). Prediction of gestational diabetes mellitus in the first trimester, comparison of fasting plasma glucose, two-step and one-step methods: a prospective randomized controlled trial. Endocrine.

[2] Shobeiri SS, Abediankenari S, Lashtoo-Aghaee B, Rahmani Z, Esmaeili-Gorji B (2016). Evaluation of soluble human leukocyte antigen-G in pripheral blood of pregnant women with gestational diabetes melitus. Caspian J Intern Med.

[3] Brogin Moreli J, Cirino Ruocco AM, Vernini JM, Rudge MV, Calderon IM (2012). Interleukin 10 and tumor necrosis factor-alpha in pregnancy: aspects of interest in clinical obstetrics. ISRN Obstet Gynecol.

[4] Vrachnis N, Belitsos P, Sifakis S (2012). Role of adipokines and other inflammatory mediators in gestational diabetesmellitus and previous gestational diabetes mellitus. Int J Endocrinol.

[5] Takhshid MA, Haem Z, Aboualizadeh F (2015). The association of circulating adiponectin and + 45 T/G polymorphism of adiponectin gene with gestational diabetes mellitus in Iranian population. J Diabetes Metab Disord.

[6] Gomes CP, Torloni MR, Gueuvoghlanian-Silva BY (2013). Cytokine levels in gestational diabetes mellitus: a systematic review of the literature. Am J Reprod Immunol.

[7] Xu J, ZhaoYH, Chen YP (2014). Maternal circulating concentrations of tumor necrosis factor-alpha, leptin, and adiponectin in gestational diabetes mellitus: a systematic review and meta-analysis. ScitificWorldJournal.

[8] Khosrowbeygi A, Ahmadvand H (2009). Maternal serum levels of adiponectin in preeclampsia. J Ayub Med Coll Abbottabad.

[9] American Diabetes Association (2014). classification of diabetes mellitus. Diabetes Care.

[10] Khosrowbeygi A, Shiamizadeh N, Taghizadeh N (2016). Maternal circulating levels of some metabolic syndrome biomarkers in gestational diabetes mellitus. Endocrine.

[11] Lubkowska A, Radecka A, Bryczkowska I (2015). Serum Adiponectin and Leptin Concentrations in Relation to Body Fat Distribution, Hematological Indices and Lipid Profile in Humans. Int J Environ Res Public Health.

[12] Friedewald WT, Levy RI, Fredrickson DS (1972). Estimation of the concentration of low-density lipoprotein cholesterol in plasma, without use of the preparative ultracentrifuge. Clin Chem.

[13] Akbas EM, Timuroglu A, Ozcicek A (2014). Association of uric acid, atherogenic index of plasma and albuminuria in diabetes mellitus. Int J Clin Exp Med.

[14] Vafaeimanesh J, Bagherzadeh M, Heidari A, Motii F, Parham M (2014). Diabetic patients infected with helicobacter pylori have a higher Insulin Resistance Degree. Caspian J Intern Med.

[15] Katz A, Nambi SS, Mather K (2000). Quantitative insulin sensitivity check index: a simple, accurate method for assessing insulin sensitivity in humans. J Clin Endocrinol Metab.

[16] López-Tinoco C, Roca M, Fernández-Deudero A (2012). Cytokine profile, metabolic syndrome and cardiovascular disease risk in women with late-onset gestational diabetes mellitus. Cytokine.

[17] Liu T, Fang Z, Yang D, Liu Q (2012). Correlation between the inflammatory factors and adipocytokines with gestational diabetes mellitus and their change in puerperium. Zhonghua Fu. Chan Ke Za Zhi.

[18] Kim SY, Sy V, Araki T (2014). Total adiponectin, but not inflammatory markers C-reactive protein, tumor necrosis factor-α, interluekin-6 and monocyte chemoattractant protein-1, correlates with increasing glucose intolerance in pregnant Chinese-Americans. J Diabetes.

[19] Mrizak I, Arfa A, Fekih M (2013). Inflammation and impaired endothelium-dependant vasodilatation in non obese women with gestational diabetes mellitus: preliminary results. Lipids Health Dis.

[20] Bao W, Baecker A, Song Y (2015). Adipokine levels during the first or early second trimester of pregnancy and subsequent risk of gestational diabetes mellitus: A systematic review. Metabolism.

[21] Al-Badri MR, Zantout MS, Azar ST (2015). The role of adipokines in gestational diabetes mellitus. Ther Adv Endocrinol Metab.

[22] Ye R, Holland WL, Gordillo R (2014). Adiponectin is essential for lipid homeostasis and survival under insulin deficiency and promotes β-cell regeneration. Elife.

[23] Li Y, Qin G, Liu J (2014). Adipose tissue regulates hepatic cholesterol metabolism via adiponectin. Life Sci.

[24] Aye IL, Rosario FJ, Powell TL, Jansson T (2015). Adiponectin supplementation in pregnant mice prevents the adverse effects of maternal obesity on placental function and fetal growth. Proc Natl Acad Sci U S A.

[25] Aye IL, Jansson T, Powell TL (2015). TNF-α stimulates System A amino acid transport in primary human trophoblast cells mediated by p38 MAPK signaling. Physiol Rep.

